# Supplementation with probiotics, prebiotics, and synbiotics in patients with chronic functional constipation: a randomized, double-blind, placebo-controlled pilot clinical trial

**DOI:** 10.1093/gastro/goae101

**Published:** 2024-11-11

**Authors:** Ana Terrén Lora, Bruno F Penadés, Sara López Oliva, Sari Arponen, Gülşah Okutan, Guerthy Melissa Sánchez Niño, Ismael San Mauro Martín

**Affiliations:** Research Centers in Nutrition and Health (CINUSA Group), Research Department, Madrid, Spain; Research Centers in Nutrition and Health (CINUSA Group), Research Department, Madrid, Spain; Research Centers in Nutrition and Health (CINUSA Group), Research Department, Madrid, Spain; Slow Medicine Institute, Research Department, Alcobendas, Madrid, Spain; Research Centers in Nutrition and Health (CINUSA Group), Research Department, Madrid, Spain; Research Centers in Nutrition and Health (CINUSA Group), Research Department, Madrid, Spain; Research Centers in Nutrition and Health (CINUSA Group), Research Department, Madrid, Spain

**Keywords:** functional constipation, probiotics, prebiotics, gastrointestinal symptoms, stool frequency, stool consistency

## Abstract

**Background:**

Functional constipation includes a set of gastrointestinal symptoms unexplainable by an identifiable underlying physical cause or pathology. The prevalence of this condition is high and there is a need to develop strategies to reduce it. Probiotics, prebiotics, and synbiotics may be an alternative treatment for chronic functional constipation.

**Methods:**

To compare the efficacy of dietary supplementation on symptoms of patients who suffer from chronic functional constipation. An exploratory, randomized, double-blind, placebo-controlled pilot clinical trial was conducted with 74 patients diagnosed with chronic functional constipation who were divided into four treatment groups—Group A: probiotics; Group B: prebiotics; Group C: synbiotics; Group D: placebo. Each patient was treated for 8 weeks. At the beginning and end of treatment, data were collected by administering questionnaires and scales, including the Bristol stool scale, on gastrointestinal symptoms, bowel movements, and sociodemographic and anthropometric characteristics.

**Results:**

Stool frequency increased in all four study groups, and greatest difference was observed in the synbiotics group (2.8 ± 1.3 vs. 5.9 ± 2.6; *P* < 0.001). Stool consistency improved only in the active treatment groups. Based on the evaluation of gastrointestinal symptoms, participants treated with prebiotics, probiotics and synbiotics showed the greatest improvement in abdominal pain (8.28 ± 2.63 vs. 6.56 ± 2.62; *P* = 0.009), gastroesophageal reflux (4.60 ± 2.66 vs. 3.45 ± 2.42; *P* = 0.039) and constipation symptoms (13.00 ± 3.97 vs. 8.71 ± 3.35; *P* = 0.003), respectively. As for quality of life, the main changes were observed in physical health domains, with a placebo effect.

**Conclusions:**

The present study provides evidence supporting the efficacy of dietary supplementation with probiotics, prebiotics, and synbiotics in patients with chronic functional constipation after 8 weeks of treatment.

## Introduction 

Functional disorders of the gastrointestinal tract encompass a set of recurrent and chronic gastrointestinal symptoms unexplainable by an identifiable underlying physical cause or pathology [1]. These functional disorders include irritable bowel syndrome (IBS) and functional constipation (FC) [2].

Broadly speaking, the term constipation is used to describe symptoms associated with defecation difficulties. These symptoms are varied and include hard or lumpy stools, infrequent bowel movements, excessive straining, a sensation of incomplete evacuation or blockage, and the use of manual maneuvers to facilitate evacuation [3]. Although difficult to quantify, the prevalence of chronic constipation is estimated at approximately 12%–19%. In addition, constipation is more common in women, in individuals with a low dietary fiber intake and in older people [4].

FC is diagnosed using Rome IV criteria [5]. The criteria defined in *Rome IV Diagnostic Criteria for Disorders of Gut-Brain Interaction* must be met within the last 3 months and the symptoms must have started within 6 months from diagnosis [2]. In addition to not meeting the criteria for diagnosis of IBS, patients must not be treated with opioids because some opioids induce constipation [3].

Currently, numerous pharmacological treatments are available for FC, including osmotic laxatives, prokinetic agents and stimulants, secretagogues, 5-HT4 receptor agonists, and peripheral opioid receptor antagonists. These pharmacological treatments are selected based on the patient’s symptoms. Other treatments include pelvic muscle training, transanal irrigation, sacral nerve stimulation, or even colon resection surgery. However, initial treatment of FC usually involves lifestyle changes (increased physical activity) and dietary changes (increased intake of water and soluble fiber) [3]. In addition to ensuring that their diet is varied and rich in soluble fiber [4], patients with FC should increase their soluble fiber intake through dietary supplementation, which is recommended by expert consensus as the first therapeutic measure [4, 6]. Yet, despite improving stool frequency and consistency, as well as straining during defecation, this therapeutic measure may also worsen abdominal pain and distension although scientific evidence of these effects remains insufficient [2]. Other studies have assessed the efficacy of probiotics in treating FC, suggesting that they regulate intestinal microbiota dysbiosis. Among all species of probiotics, *Bifidobacterium lactis* may be the most effective in improving FC symptoms. Nevertheless, supplements with multispecies probiotics seem to be more beneficial than supplements with single-species probiotics [7].

The present study aimed at assessing the efficacy of dietary supplementation with probiotics, prebiotics and synbiotics compared with placebo in improving stool frequency and consistency and symptoms of patients with FC.

## Materials and methods

### Design and participants

**Figure 1.**
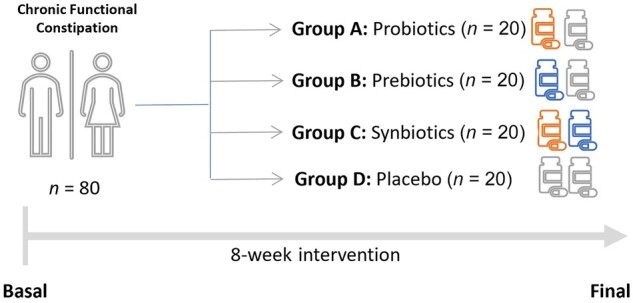
Study design.

An exploratory, randomized, double-blind, placebo-controlled pilot clinical trial (Figure 1) was conducted at an outpatient clinic of the Medical Center Paseo de la Habana, Ruber International Hospital, Research Centers in Nutrition & Health (Madrid, Spain) from January 2023 to July 2023.

The following inclusion criteria were applied in this study: (i) aged between 18 and 65 years old; (ii) body mass index 18.5–39.9 kg/m^2^; (iii) diagnosed with FC according to Rome IV criteria; and (iv) patients who signed the informed consent form and were able to understand and perform the study procedures. The female participants were required to meet one of two conditions: (i) women unable to become pregnant or (ii) women able to become pregnant who used a highly effective contraception method during the study. The exclusion criteria were as follows: (i) diagnosed with other chronic diseases (Alzheimer, Parkinson’s disease or dementia, cerebral palsy, myocardial infarction, tetraplegia, hemiplegia, intellectual disability, multiple sclerosis, amyotrophic lateral sclerosis, cystic fibrosis, congenital metabolic diseases, or eating disorders); (ii) with a history of gastrointestinal surgery; (iii) scheduled for surgery during the study; (iv) receiving anticoagulants or opioid pain relievers; (v) treated with laxatives within 15 days prior to study entry; (vi) treated with antibiotics within a month prior to study entry; (vii) consumed probiotics or prebiotics within 2 weeks prior to study entry; (viii) diagnosed with or who met sufficient criteria for a diagnosis of ulcerative colitis, Crohn’s disease, intestinal obstruction or gastrointestinal malignancy; (ix) enrolled in another clinical trial with a pharmaceutical drug within 30 days prior to study entry; (x) pregnant or lactating women; or (xi) patients with hypersensitivity, allergy or intolerance to any component of the food supplements under study.

All procedures followed were in accordance with the ethical standards of the responsible committee on human experimentation (institutional and national) and with the Helsinki Declaration of 1964 and subsequent versions. This study was reviewed and approved by the Drug Research Ethics Committee of Quirónsalud Hospital Group (Catalunya, Spain), with the reference: ESTR-01-2022 (2022/86-NUT-CEX; CEIm approval no. 23/2022), on 23 November 2022. Each participant was verbally informed about the study and signed the informed consent form before undergoing the study procedures. The trial registration number (ClinicalTrials.gov) was NCT06381193.

### Randomization and blinding

Randomization was performed by an independent statistician, who generated a list of treatment kits assigned to each participant. On the first study visit (Day 0), the research team provided each subject included in the study with the treatment kits. The different treatments were packaged and labeled in the same way and looked identical. As this was a double-blind study, both researchers and participants were unaware of the assigned treatment until the end of the study.

### Sample size

No data on the same probiotics or synbiotics were available for the study population. For this reason, an exploratory pilot study was planned according to the resources assigned for such purpose.

### Intervention

All participants were randomized to one of the four study groups—Group A: probiotics formulated with four bifidobacterial strains (*B. breve* BB03, *B. lactis* BL04, *B. longum* BL05 and *B. bifidum* BB06); Group B: prebiotics based on oat fiber, inulin, fructooligosaccharides and marshmallow dry extract; Group C: synbiotics; Group D: maltodextrin-based placebo. Each patient received the treatment (divided into two bottles) needed for 8 weeks. During this period, the patients took four units of product per day (two units from each bottle) with a large glass of water after one of the main meals of the day.

At each visit, measurements were taken, and the corresponding questionnaires were administered. Similarly, data on adverse events and medication used during the study was recorded at the last visit.

### Questionnaires and data collection

In addition to the primary outcome (stool frequency), secondary outcomes were assessed after 8 weeks of treatment based on the following: stool consistency [Bristol stool scale (BSS)] [8] and the quality of life specific questionnaire for constipated patients 20 (CVE-20) [9] and Gastrointestinal Symptom Rating Scale (GSRS) scores [10].

Demographic, lifestyle, and medical history data were collected during the participant selection visit. Stool consistency and frequency data were collected in both study visits. Stool consistency was measured using the BSS. In addition, CVE-20 and GSRS were administered in both visits. Weight was measured on a tetrapolar bioimpedance analyzer (TANITA model BP-601), and waist circumference was measured using an anthropometric tape measure (Cescorf).

### Statistical analysis

Patient characteristics were described before the intervention, assessing potentially significant differences between supplementation groups using the unpaired Student’s *t*-test or the Mann–Whitney *U* test for numerical variables with a normal distribution or the Shapiro–Wilk test for numerical variables without a normal distribution. In turn, Fisher’s exact test was used for categorical variables.

To detect whether patient-reported scores varied with the visit times, the paired Student’s *t*-test was performed, using the Wilcoxon signed-rank test as a nonparametric alternative whenever the assumption of normality was not satisfied and the sign test whenever the assumption of symmetry was not satisfied. Lastly, the data of each group were transformed into delta (Δ) values, that is, the difference between the visits of each patient, to compare groups using the unpaired Student’s *t*-test or the Mann–Whitney *U* test as a nonparametric alternative given the lack of normality in any group.

The data are expressed as mean and standard deviation. Pairwise comparisons between hypotheses were performed with a confidence level of 95% in the R software environment for statistical computing and graphics (version 4.1.3).

## Results

### Sample and patient characteristics

**Figure 2.**
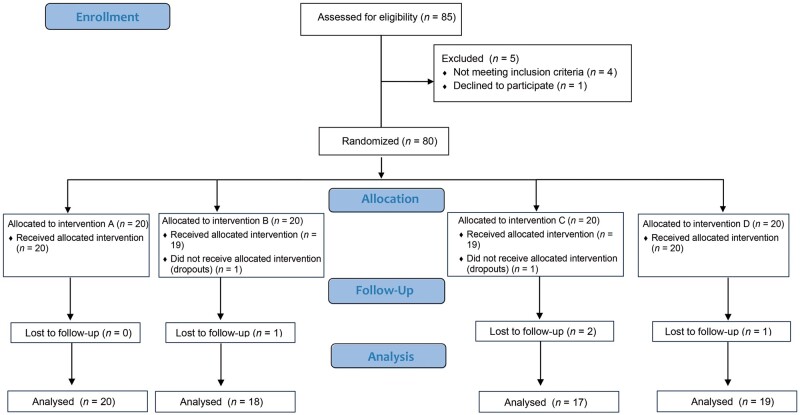
Participant flowchart.

A total of 85 patients were enrolled, 80 of whom met the inclusion criteria. Of the 80 included subjects, 74 completed the study (Figure 2). The sample consisted of 71 females and 3 males with a mean age of 45.5 ± 10.9 years. Most patients were Caucasian (93.2%), followed by Latinos (6.8%). A preliminary analysis of preintervention patient characteristics showed no differences between the study groups (Table 1).

**Table 1.** Patient characteristics before the intervention

Characteristic	Probiotics (*n* = 20)	Prebiotics (*n* = 18)	Synbiotics (*n* = 17)	Placebo (*n* = 19)	*P*-value
Demographic data					
Age	44.9 ± 9.3	47.0 ± 8.2	48.3 ± 11.9	42.3 ± 13.3	0.364^a^
Female	18 (90.0)	18 (100)	16 (94.1)	19 (100)	0.450^c^
Ethnicity					0.805^c^
Caucasian	19 (95.0)	17 (94.4)	15 (88.2)	18 (94.7)	
Latino	1 (5.0)	1 (5.6)	2 (11.8)	1 (5.3)	
Anthropometric data					
Weight, kg	62.6 ± 11.1	64.2 ± 13.7	63.7 ± 17.2	60.5 ± 9.2	0.937^b^
Height, m	1.68 ± 0.06	1.63 ± 0.06	1.63 ± 0.08	1.66 ± 0.05	0.05^a^
BMI, kg/m^2^	22.1 ± 3.7	24.2 ± 5.2	23.7 ± 5.8	22.0 ± 3.1	0.548^b^
Waist circumference, cm	73.9 ± 8.0	77.2 ± 13.1	78.7 ± 13.4	72.7 ± 7.6	0.576^b^
Lifestyle habits					
MEDAS	9.40 ± 1.93	9.39 ± 1.75	9.59 ± 1.46	8.68 ± 2.16	0.680^b^
IPAQ	1918.66 ± 990	1402.97 ± 1117.97	1480.47 ± 1021.61	1429.53 ± 1271.97	0.231^b^
Sleep quality					
Weekday sleep, hours	7.08 ± 0.88	6.72 ± 1.00	6.74 ± 1.63	7.11 ± 1.24	0.615^b^
Weekend sleep, hours	7.63 ± 1.38	7.67 ± 1.54	7.41 ± 1.95	7.84 ± 1.33	0.676^b^

The results are expressed as mean ± standard deviation or frequency and percentage (%).

a Analysis of variance.

b Kruskal–Wallis test.

c Fisher’s exact test.

BMI = body mass index, MEDAS = Mediterranean Diet Adherence Screener, IPAQ = International Physical Activity Questionnaire.

### Stool frequency and consistency

**Figure 3.**
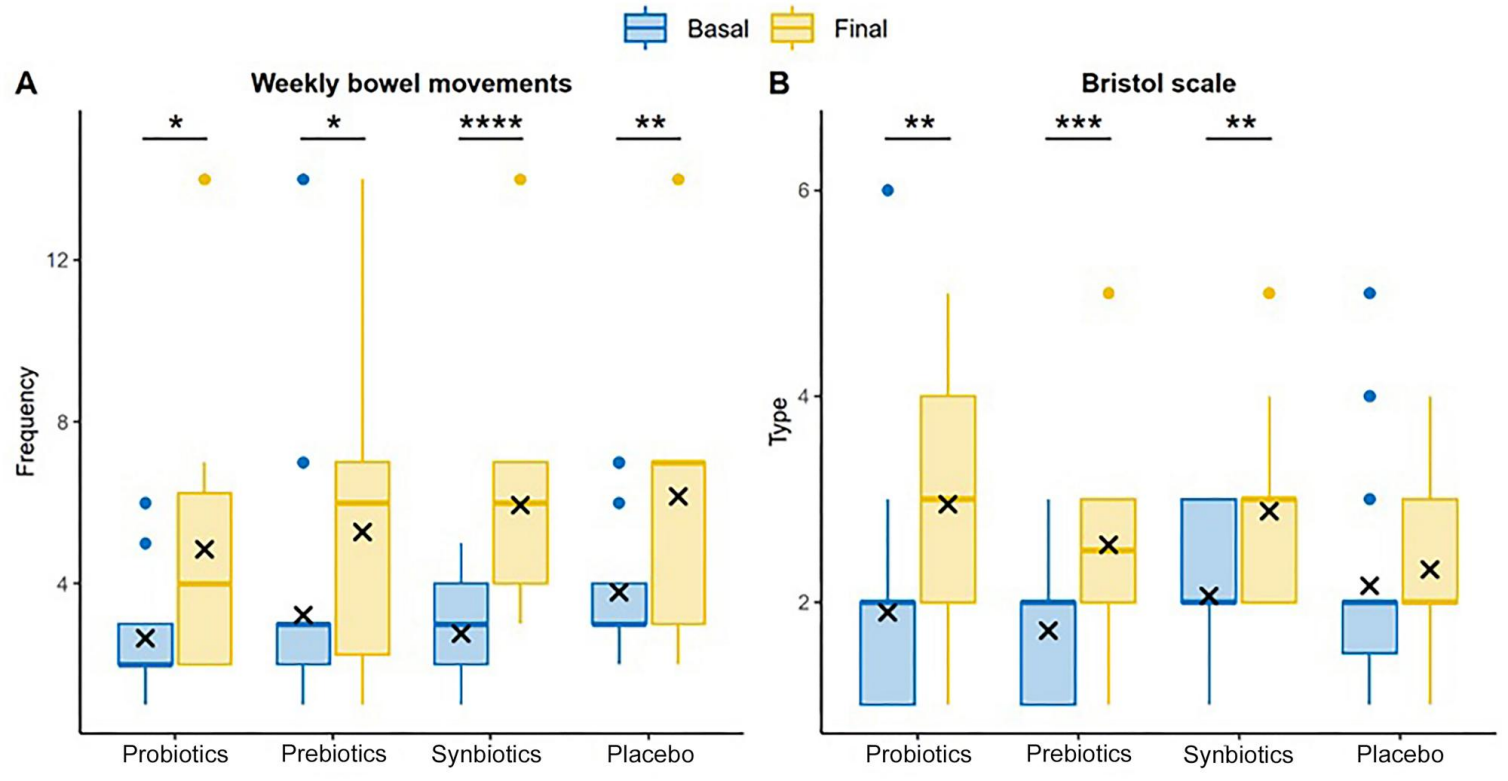
Comparison of stool frequency and consistency results in four groups. (A) Weekly stool frequency. (B) Type of stool consistency according to the Bristol stool scale. Results are expressed as means recorded in each visit. *P < 0.05, **P < 0.01, ***P < 0.001, ****P < 0.0001.

Patient-reported weekly stool frequency increased in all four study groups after 8 weeks of dietary supplementation (Figure 3A). This change was clearly observed in the synbiotics group (2.8 ± 1.3 vs. 5.9 ± 2.6; *P* < 0.001). However, no significant differences were detected when comparing intergroup differences before and after the intervention (Table 2).

**Table 2.** Stool assessment based on CVE-20 and GSRS

Item	Probiotic (*n* = 20)			Prebiotic (*n* = 18)			Synbiotics (*n* = 17)			Placebo (*n* = 19)			Δ
	Start	Final	*P*	Start	Final	*P*	Start	Final	*P*	Start	Final	*P*	*P*
Feces													
Weekly frequency	2.7 ± 1.5	4.9 ± 3.6	**0.013** ^c^	3.2 ± 3.0	5.3 ± 3.2	**0.017** ^a^	2.8 ± 1.3	5.9 ± 2.6	**<0.001** ^c^	3.8 ± 1.7	6.2 ± 3.3	**0.005** ^a^	0.529^e^
Bristol scale	1.90 ± 1.21	2.95 ± 1.23	**0.008** ^a^	1.72 ± 0.58	2.56 ± 1.15	**0.000** ^c^	2.06 ± 0.75	2.88 ± 0.93	**0.004** ^a^	2.16 ± 1.12	2.32 ± 0.95	0.461^b^	0.202^e^
Quality of life specific questionnaire for constipated patients 20 (CVE-20)													
Emotional dimension	13.85 ± 4.89	14.65 ± 5.01	0.374^a^	13.44 ± 4.13	15.44 ± 4.90	0.090^a^	14.18 ± 5.03	15.76 ± 4.18	0.146^c^	13.26 ± 4.64	14.79 ± 5.33	0.068^b^	0.891^e^
General physical dimension	11.30 ± 5.27	13.45 ± 5.78	**0.034** ^a^	10.89 ± 3.64	12.89 ± 5.10	**0.039** ^a^	11.53 ± 3.20	15.35 ± 3.79	**0.001** ^a^	12.21 ± 3.94	14.79 ± 5.00	0.064^c^	0.312^e^
Rectal physical dimension	7.50 ± 3.30	9.20 ± 4.07	**0.039** ^a^	8.00 ± 2.81	10.50 ± 3.22	**<0.001** ^c^	9.88 ± 2.85	11.06 ± 3.53	**0.022** ^a^	9.74 ± 3.87	11.53 ± 3.55	**0.039** ^a^	0.674^e^
Social dimension	5.40 ± 3.44	6.80 ± 3.32	0.146^c^	5.78 ± 2.21	6.56 ± 3.05	0.454^c^	6.94 ± 2.36	7.71 ± 1.99	0.314^a^	6.79 ± 2.25	7.84 ± 2.24	**0.043** ^b^	0.717^e^
Gastrointestinal Symptom Rating Scale (GSRS)													
Abdominal pain	7.95 ± 3.94	6.20 ± 2.55	0.058^c^	8.28 ± 2.63	6.56 ± 2.62	**0.009** ^a^	8.00 ± 3.04	7.47 ± 4.00	1.000^c^	8.58 ± 4.51	7.00 ± 3.43	0.074^a^	0.855^e^
Reflux	4.60 ± 2.66	3.45 ± 2.42	**0.039** ^c^	3.33 ± 2.11	3.06 ± 1.39	0.562^a^	3.88 ± 1.90	4.12 ± 2.69	1.000^c^	3.47 ± 1.93	3.53 ± 2.86	0.754^c^	0.428^e^
Diarrhea	5.70 ± 3.13	4.90 ± 2.88	0.214^b^	4.83 ± 2.20	5.17 ± 2.77	0.607^a^	5.94 ± 3.46	7.06 ± 4.51	0.804^c^	5.79 ± 3.65	5.21 ± 2.70	0.791^c^	0.458^e^
Indigestion	14.85 ± 6.15	11.25 ± 5.24	**0.004** ^a^	14.50 ± 4.76	12.56 ± 4.72	**0.035** ^a^	13.88 ± 3.81	10.35 ± 3.64	**0.002** ^a^	14.89 ± 4.89	11.79 ± 4.93	**0.015** ^a^	0.654^d^
Constipation	14.60 ± 3.55	11.15 ± 4.80	**0.025** ^a^	14.78 ± 3.95	12.00 ± 4.49	**0.009** ^a^	13.00 ± 3.97	8.71 ± 3.35	**0.003** ^a^	14.16 ± 3.11	12.00 ± 5.18	0.064^a^	0.633^d^

The results are expressed as mean ± standard deviation. Deltas (Δ), difference for start-final. Values in bold are statistically significant results.

a Paired Student’s *t*-test.

b Wilcoxon signed-rank test.

c Sign test.

d Analysis of variance.

e Kruskal–Wallis test.

CVE-20 = quality of life specific questionnaire for constipated patients 20, GSRS = Gastrointestinal Symptom Rating Scale.

As for stool consistency, measured using the BSS, all participants, except for those assigned to the placebo group, reported that their stools were less consistent after dietary supplementation. Thus, significant differences were found when comparing data from the prebiotics (1.72 ± 0.58 vs. 2.56 ± 1.15; *P* < 0.001), probiotics (1.90 ± 1.21 vs. 2.95 ± 1.23; *P* = 0.008) and synbiotics (2.03 ± 0.75 vs. 2.88 ± 0.93; *P* = 0.004) groups between the first and last visit (Figure 3B).

### Quality of life according to the CVE scale

**Figure 4.**
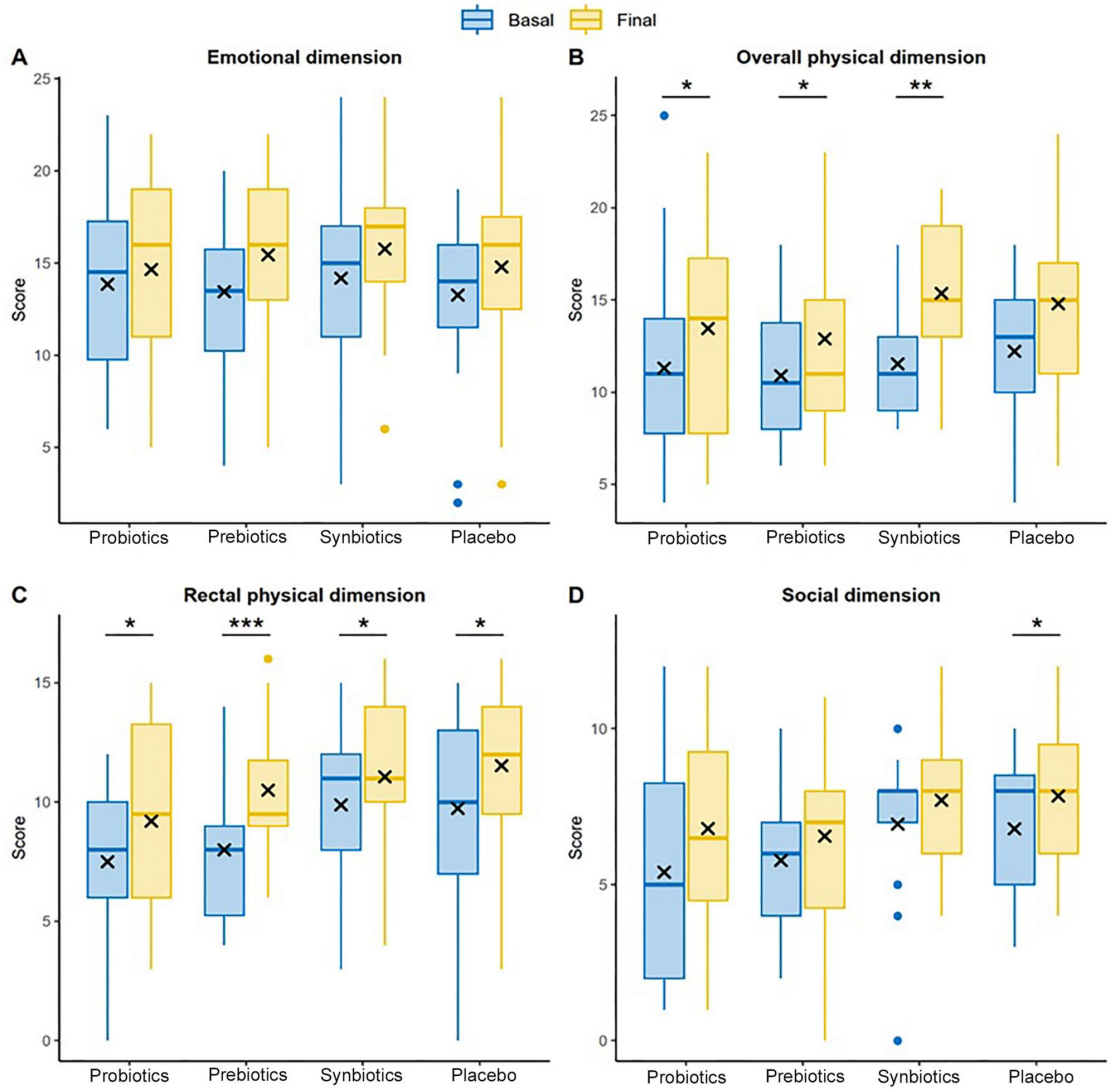
Comparison of the quality of life specific questionnaire for constipated patients 20 score in four groups. (A) Scores of the psychological health domain. (B) Scores of the general physical health domain. (C) Scores of the rectal physical health domain. (D) Scores of the social relationships domain. The results are expressed as means recorded in each visit. *P < 0.05, **P < 0.01, ***P < 0.001.

The quality of life of patients with FC was evaluated based on four domains, according to the CVE. First, positive trends were observed in the psychological health domain, especially in the prebiotics and synbiotics groups, albeit nonsignificant (Figure 4A). Second, the general physical health domain improved in all four groups after supplementation, but the change in the placebo group was not significant (Figure 4B). Third, the rectal physical health domain improved in all groups, especially in the prebiotics group (8.00 ± 2.81 vs. 10.50 ± 3.22; *P* < 0.001), whose results were highly significant (Figure 4C). Finally, the pattern observed in the social relationship domain also improved in the study groups after 8 weeks of supplementation, with the placebo group slightly standing out from the other groups (6.79 ± 2.25 vs. 7.84 ± 2.24; *P* = 0.043) (Figure 4D).

In all groups, quality of life improved across all domains, but significant changes were concentrated in the physical health domains and in the treatment groups (Table 2).

### GSRS

**Figure 5.**
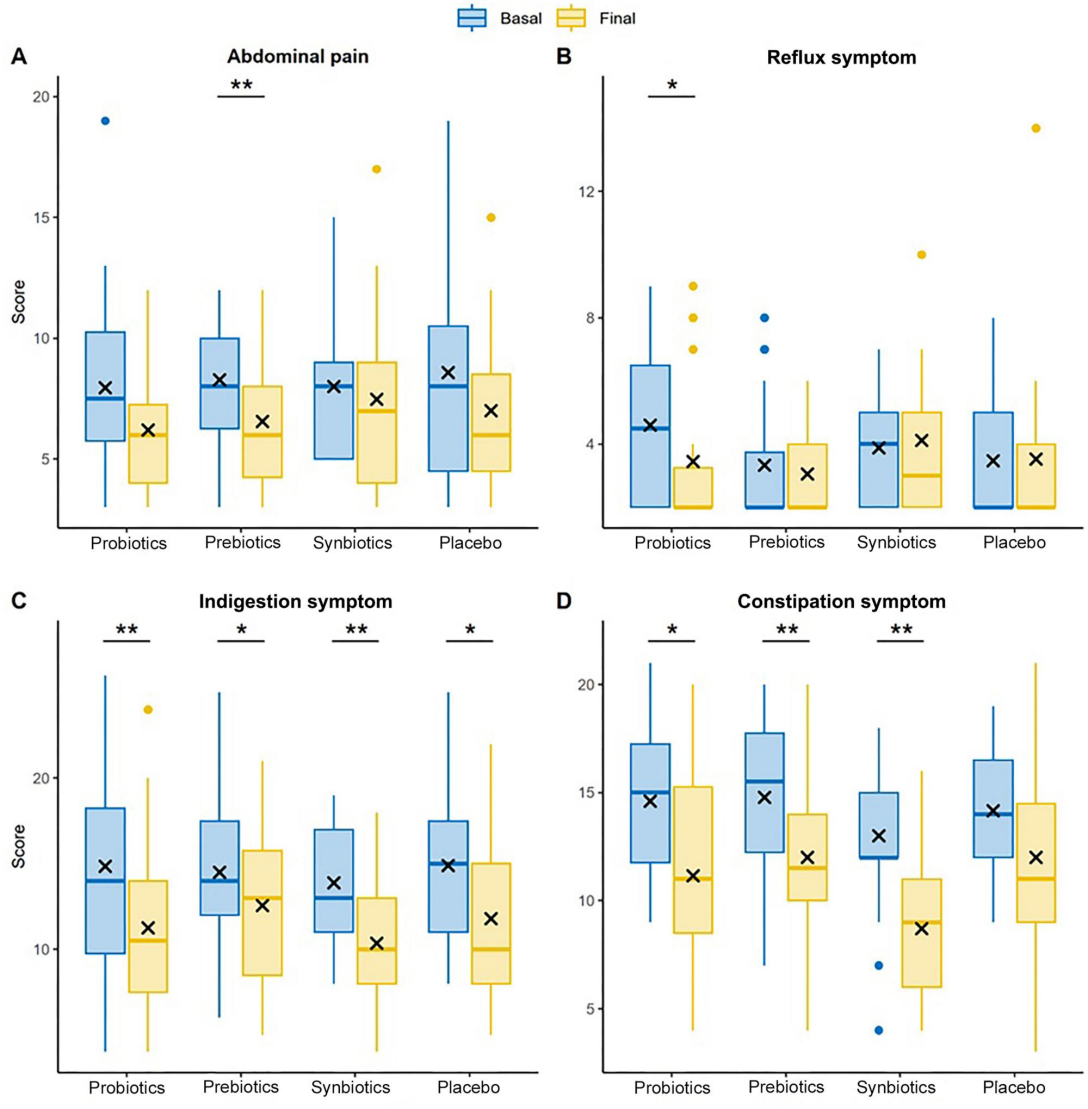
Comparison of Gastrointestinal Symptom Rating Scale score in four groups. (A) Scores of abdominal pain. (B) Scores of gastroesophageal reflux symptoms. (C) Scores of indigestion symptoms. (D) Scores of constipation symptoms. The results are expressed as means recorded in each visit. *P < 0.05, **P < 0.01.

Participant’s quality of life was also examined using GSRS. Among the factors analyzed in this scale, abdominal pain significantly decreased in the prebiotics group (8.28 ± 2.63 vs. 6.56 ± 2.62; *P* = 0.009), although the remaining patients assigned to the other groups also reported lower scores (Figure 5A). In turn, all groups remained relatively stable regarding gastroesophageal reflux symptoms, except for the probiotics groups, which showed a significant difference in scores after 8 weeks of supplementation (4.60 ± 2.66 vs. 3.45 ± 2.42; *P* = 0.039) (Figure 5B). Indigestion symptoms improved in all groups, albeit to a greater extent in the probiotics (14.85 ± 6.15 vs. 11.25 ± 5.24; *P* = 0.004) and synbiotics (13.88 ± 3.81 vs. 10.35 ± 3.64; *P* = 0.002) groups (Figure 5C). Constipation scores decreased in all treatment groups, but not in the placebo group, that is, only patients who consumed probiotics (14.60 ± 3.55 vs. 11.15 ± 4.80; *P* = 0.025), prebiotics (14.78 ± 3.95 vs. 12.00 ± 4.49; *P* = 0.009) or synbiotics (13.00 ± 3.97 vs. 8.71 ± 3.35; *P* = 0.003) noticed significant improvements in their constipation symptoms (Figure 5D).

## Discussion

The present clinical trial showed evidence supporting the efficacy of dietary supplementation with probiotics, prebiotics, and synbiotics in treating patients with chronic FC after 8 weeks of intervention.

All participants reported an increased weekly stool frequency after the intervention, with the synbiotics group showing the greatest change, but with no significant differences between groups, as in previous studies. For example, stool frequency increased after treatment with both probiotics and a placebo, to a larger extent in the probiotics group, albeit without significant differences between the two groups [11]. Lai *et al.* [12] reached similar conclusions as all treatment groups, including placebo, increased the intestinal evacuation frequency. Their results corroborated the findings of Martoni *et al.* [13], who did not observe significant differences between groups (probiotics vs. placebo) either although the trend was more positive in the probiotics group.

Notwithstanding the above, a meta-analysis by Miller *et al.* [14] showed that stool frequency was significantly higher in probiotics groups than in placebo groups in 11 of the 20 comparisons. A compilation of several studies on dietary supplementation with prebiotics (especially inulin) for treating constipation showed that prebiotics increased stool frequency [15], as reported in the meta-analysis by van der Schoot *et al.* [16]. However, other studies did not find significant differences between probiotics and conventional treatments with fiber because stool frequency increased in all cases [17]. Despite these discrepancies, treatment with synbiotics has been shown to decrease colonic transit time, increase stool frequency, and improve stool consistency [15].

The placebo effect observed during the present study may explain why some results were not significant. In fact, in patient-reported outcome studies on treatment with probiotics, the placebo effect seems to be frequent [18]. Nevertheless, supplementation with synbiotics may be the best strategy for increasing stool frequency. On the one hand, supplementation with synbiotics includes the potential effect of probiotics on intestinal motility by modulating gut–brain axis microbiota and, therefore, the activity of the central and enteric nervous systems [19] and by increasing mucin secretion, which facilitates peristalsis. On the other hand, this treatment also includes prebiotics, such as inulin, which also modulate the intestinal microbiota, stimulating the proliferation of commensal bacteria that produce short-chain fatty acids, such as butyrate. Among other functions, these short-chain fatty acids seem to stimulate intestinal motility [15]. Therefore, treatment with synbiotics may produce a synergistic effect, so the benefits of both treatments may be leveraged, although further studies are needed to confirm this potential.

In our study, stool consistency improved in all active treatment groups, whereas the placebo group showed no significant improvement at the end of the study. This positive effect seems to be a recurring trend in other studies, especially when the treatment includes a probiotic. In fact, this effect usually appears a few days after treatment, as shown by Martoni *et al.* [13]. In the study conducted by these authors, stool consistency was significantly better in the group treated with probiotics for only one week of intervention than in the placebo group. These data are also supported by a meta-analysis published in 2020, which determined that dietary supplementation with probiotics, especially with several species, improves stool frequency and consistency and gut transit time [7].

In individuals with low stool frequency (≤3/week), BSS scores increased and straining during defecation decreased after treatment with a high dose of probiotics [20]. Similarly, not only probiotics improve FC, but prebiotics may also play a key role in stool consistency. Dietary supplementation with prebiotics, particularly *Psyllium* and inulin, significantly improves stool consistency in patients with chronic constipation [16].

As recently shown, stool consistency improves in individuals treated with prebiotics or probiotics, but not when administering a placebo [12]. These findings are in line with the results of the present study. Nevertheless, some studies have indicated that stool consistency improves primarily when using synbiotics [15], whereas other studies have indicated that dietary supplementation with probiotics does not significantly improve stool consistency compared with placebo or another supplement [17, 18]. This controversy could be due to the heterogeneity among studies in terms of the type of probiotics and prebiotics, their doses, and treatment time.

Quality of life assessed using the CVE-20 questionnaire improved relatively in at least one of the domains in all study groups. The most significant changes were observed in the active treatment groups and in the physical health domain. In the social relationship domain, the placebo group slightly stood out from the other groups possibly because the corresponding scores already stood out before the placebo treatment. Other studies have not identified significant differences in quality of life scores between individuals with constipation undergoing dietary supplementation with fiber [16] or probiotics [13, 18, 20] and individuals treated with a placebo.

The progression of symptoms was also examined using the GSRS. Among the factors evaluated in the participants, abdominal pain significantly decreased in the prebiotics group, and gastroesophageal reflux symptoms significantly improved in the probiotics group. By contrast, indigestion symptoms improved in all groups, albeit to a greater extent in the probiotics and synbiotics groups. In turn, constipation symptoms only improved in patients under active treatment. Along these lines, Bayer *et al.* [21] observed that dietary supplementation with soluble kiwi or *Psyllium* fiber significantly improved the GSRS scores of patients with FC or IBS with constipation, in both groups after the intervention. Based on the combined results from several studies, supplementation with fiber is effective in improving symptoms associated with constipation only from a dosage of 10 g per day for 4 weeks [16].

Symptoms such as bloating or belching seem to improve significantly after supplementation with probiotics compared with placebo, but no significant differences were found in other symptoms, including flatulence [22]. Nevertheless, another study demonstrated an improvement in all GSRS categories after treatment with probiotics when compared with the placebo group. In this case, the most important differences were observed in the subcategories bloating, belching, and flatulence [23]. Conversely, the results from a meta-analysis showed that abdominal bloating did not improve after treatment with either probiotics or placebo [7]. Yet, in a clinical trial conducted by Lai *et al.* [12], straining during defecation decreased in all participants. Accordingly, there is no clear consensus on the effect of treatment with synbiotics on constipation-related symptoms, such as bloating and abdominal pain [15].

This study has some limitations. First, neither the subtype of constipation nor previous failed treatments of each participant were classified, which could have provided clues about their response to treatment. Although the study participants met the inclusion criteria described in the protocol, some of them tended to use products to facilitate evacuation, such as laxatives, enemas, and dietary or nutritional supplements (such as seeds or infusions) on a more or less regular basis. This circumstance was overlooked when assessing the impact of changes in therapy or its synergistic effect. Second, the baseline patient characteristics varied widely in terms of type of diet and physical exercise. And while the total sample size was considerable, the treatment subgroups were relatively small, which may explain difficulties in identifying significant differences between groups.

## Conclusions

The present study provides preliminary evidence supporting the efficacy of dietary supplementation with probiotics, prebiotics, and synbiotics, in patients with chronic FC after 8 weeks of treatment. Combined, these encouraging results highlight the potential benefits of this treatment for individuals suffering from chronic FC although further research must be conducted to improve protocols.

## Authors’ Contributions

I.S.M.M. and S.A. were responsible for conceptualization. I.S.M.M. was responsible for funding acquisition and resources. A.T.L., G.O., and I.S.M.M. were responsible for investigation. A.T.L. and I.S.M.M. were responsible for methodology, project administration, and supervision. A.T.L. was responsible for validation and visualization. B.F.P. was responsible for data curation. B.F.P. and I.S.M.M. were responsible for formal analysis. A.T.L., B.F.P., S.L.O., S.A., G.O., G.M.S.N., and I.S.M.M were responsible for drafting, revising, and editing of the manuscript. All authors read and approved the final version of the manuscript.
